# BCL-W expression associates with poor outcome in patients with peripheral T-cell lymphoma not otherwise specified

**DOI:** 10.1038/s41408-021-00549-6

**Published:** 2021-09-16

**Authors:** Mario L. Marques-Piubelli, Luisa M. Solis, Edwin R. Parra, Luis Malpica Castillo, Sushanth Gouni, Ranjit Nair, Dai Chihara, Marina Konopleva, Ignacio I. Wistuba, Swaminathan P. Iyer, Francisco Vega, Paolo Strati

**Affiliations:** 1 Department of Translational Molecular Pathology, The University of Texas MD Anderson Cancer Center, Houston, TX USA; 2 Department of Lymphoma and Myeloma, The University of Texas MD Anderson Cancer Center, Houston, TX USA; 3 Department of Leukemia, The University of Texas MD Anderson Cancer Center, Houston, TX USA; 4 Department of Hematopathology, The University of Texas MD Anderson Cancer Center, Houston, TX USA

**Keywords:** Translational research, Apoptosis

**To the Editor:**

The pro-survival BCL-2 family members BCL-2, BCL-XL (BCL2L1), BCL-W (BCL2L2), BCL-2A1 and MCL-1 contribute to tumor maintenance, progression, and chemo-resistance across a range of cancers, but their contributions in Peripheral T-cell Lymphoma Not Otherwise Specified (PTCL-NOS) are poorly understood [1]. BH3 profiling, is today considered the gold standard for prediction of BCL-2 family proteins dependence. However, it can be performed only on fresh material and requires major expertise, available in only in limited centers [2, 3].

Direct evaluation of BCL-2 alternative protein expression by immunohistochemistry (IHC) is a more commonly available approach, and can be performed on paraffin embedded samples [4]. In a series of 112 patients, each protein was detected by IHC in a subset of PTCLs, using a 10% cut-off, with variable expression based on PTCL subtypes (median, BCL-2 46%, BCL-XL 57%, and MCL-1 65%) [5]. In a series of 173 patients with PTCL, BCL-2 expression was confirmed by IHC in 64% of cases, again with extreme variability across PTCL subtypes [6]. Therefore, the prognostic role of BCL-2 expression in PTCL has been only partially explored, with conflicting reported data, and limited information regarding the prognostic role of other BCL-2 family proteins [7, 8].

This is a retrospective analysis of patients with PTCL-NOS receiving either frontline or salvage therapy at the MD Anderson Cancer Center between 09/2000 and 09/2019, and for whom pre-treatment tissue biopsy samples were available for correlative analysis. The 2014 Lugano Classification was retrospectively applied to determine treatment response in all patients [9]. The study was approved by the Institutional Review Board of The University of Texas MD Anderson Cancer Center and was conducted in accordance with the principles of the Declaration of Helsinki.

After routine diagnostic assessment, formalin-fixed paraffin embedded (FFPE) of lymph node and extranodal tissues were sectioned at a thickeness of 4 μm and stained using Leica Bond RX automated stainer (Leica Biosystems). Primary antibody against BCL-2 (clone E17, cat# ab32124, dilution 1:200), MCL-1 (clone D5V5L, cat#39224 S, dilution 1:100), BCL-W (polyclonal, cat# AF8241, dilution 1:50), BCL-2A1 (polyclonal, cat# ab45413, dilution 1:200), BCL-XL (clone 54H6, cat# 2764, dilution 1:1500), Tbet (TBX21/Tbet, clone 4B10, cat# ab91109, dilution 1:300), CXCR3 (clone 1C6/CXCR3, cat# 557183, dilution 1:200), GATA3 (clone EPR166551, cat# ab199428, concentration 1:250), and CCR4 (polyclonal, cat# HPA031613, dilution 1:200) were used and incubated for 15 min at room temperature. The antibody was detected using Bond Polymer Refine Detection kit (Leica Biosystems) with Diaminobenzidine (DAB) as chromogen. Tonsils were used as external positive controls and reactive immune cells were used as internal control. All cases were analyzed using standad microscopy by three expert hemato-pathologists. The BCL-2 family proteins scoring was assessed based on percentage of positive neoplastic cells (whole slide) and scored in 10% increments. The cell-of-origin (COO) for PTCL-NOS (TBX21/Th1 *versus* GATA3/Th2 subgroup) was assessed using the algorithm proposed by Amador et al. [10].

Association with categorical variables was evaluated using χ2 or Fisher exact tests, as appropriate. The difference in a continuous variable between patient groups was evaluated by the Mann-Whitney test. Progression-free survival (PFS) was defined as time from start of therapy to progression of disease, death, or last follow-up (whichever occurred first), and censored at time fo stem cell transplant. OS was defined as time from start of therapy to death or last follow-up. PFS and OS were calculated for all patients in the study and for subgroups of patients using Kaplan–Meier estimates, and were compared between subgroups using the log rank test. Multivariable Cox regression analysis was performed to assess the associations between patient characteristics and PFS or OS. A *p*-value of ≤0.05 (two-tailed) was considered statistically significant. Statistical analyses were completed using SPSS 21 and GraphPad Prism 8.

Twenty-seven patients with PTCL-NOS were included in the study, with a median age of 65 years (range, 22–87 years). Sixteen (59%) patients were previously treated, with a median number of 2 systemic therapies (range, 1–7), including SCT in 5 (19%; autologous in 4, allogeneic in 1 patient). Remaining baseline characteristics are shown in Table 1.

**Table 1:** Baseline characteristics of the patients with PTCL-NOS.

Patients (N = 27)	Number (%), median [range]
**Age** **<** **65 years**	13 (48)
**≥** **65 years**	14 (52)
**Female**	9 (23)
**Male**	18 (67)
**Stage I-III**	14 (52)
**IV**	13 (48)
**Previously untreated**	11 (41)
**Previously treated**	16 (59)
**Previous therapies** **<** **2**	16 (59)
**≥** **2**	11 (41)
**No previous SCT**	22 (81)
**Previous SCT**	5 (19)
**No previous XRT**	25 (93)
**Previous XRT**	2 (7)
**Th1 phenotype**	15 (56)
**Th2 phenotype**	10 (37)
**Unclassified**	2 (7)
**MCL-1 (%)**	70 [1–100]
**BCL-W (%)**	100 [40–100]
**BCL-XL (%)**	10 [0–90]
**BCL-2A1 (%)**	20 [0–90]
**BCL-2 (%)**	30 [1–100]

*SCT* stem cell transplant, *XRT* radiotherapy, *Th* T helper.

COO and BCL-2 family proteins expression were assessed on all pre-treatment tissue samples by IHC. Fifteen (56%) patients showed a TBX21/Th1 phenotype, 10 (37%) GATA3/Th2 and 2 (7%) were unclassifiable (Supplementary Fig. 1). The median expression of BCL-2, BCL-XL, BCL-W, BCL-2A1 and MCL-1 was 30% (range: 0–90%), 10% (range: 0–90%), 100% (range: 40–100%), 20% (range: 0–90%), and 70% (range: 1–100%), respectively (Supplementary Fig. 2). Of interest, only BCL-2 and BCL-W were expressed in 100% of cells, in 11 and 67% of cases, respectively. BCL-2A1 expression was significantly higher in patients who were previously treated (median, 35% vs 5%, *p* = 0.02), and in those who had previously received 2 or more lines of therapy (median, 40% vs 5%, *p* = 0.02); BCL-2 expression was significantly higher in patients with a GATA3/Th2 phenotype (60% vs 20%, *p* = 0.05)(Fig. 1A–E). No significant difference in other BCL-2 family proteins expression was observed according to baseline characteristics.

**Fig. 1:**
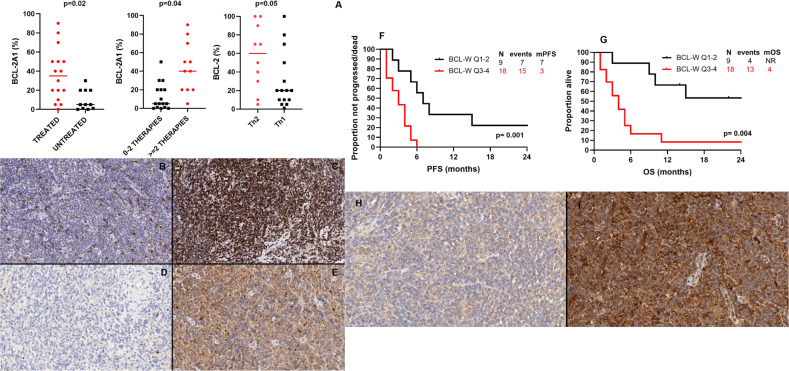
Association between BCL-2 family proteins expression and baseline characteristics, and survival according to BCL-W expression. **A** Association between BCL-2A1 levels and previous therapy, and BCL-2 lvels and Th2 phenotype. **B** BCL-2 immunostain shows strong positivity in only 10% of lymphoma cells (low expression). **C** BCL-2 immunostain shows strong expression in 100% of lymphoma cells (high expression). **D** BCL-2A1 immunostain shows negativity in all lymphoma cells (low expression). **E** BCL-2A1 immunostain shows diffuse and moderate to strong cytoplasmic expression in 80% of lymphoma cells (high expression). **F** Progression-free survival according to BCL-W expression. **G** Overall survival according to BCL-W expression. **H** BCL-W immunostain shows weak cytoplasmic positivity in 40% of lymphoma cells (low expression). **I** BCL-W immunostain shows diffuse and intense expression in 100% of lymphoma cells (high expression).

Among the 27 patients included in this study, 24 (89%) received treatment after tissue biopsy collection, including chemotherapy in 17 (63%) and biological therapy in 7 (16%), for a median of 2 cycles (range, 1–6); 3 (11%) did not receive any further treatment. Overall, 19 (76%) patients were evaluable for response, whereas 5 (24%) died or discontinued subsequent therapy (due to toxicity) before first response assessment. CR was achieved in 5 (24%) patients, and 6 (22%) patients proceeded with subsequent consolidation with SCT, including autologous SCT in 4 patients and allogeneic in 2. A trend for higher BCL-W expression was observed among patients not achieving CR (100% vs 90%, *p* = 0.07). No significant difference in BCL-2 family proteins expression was observed when comparing patients who achieved CR to those who did not.

After a median follow-up of 28 months (95% confidence interval [CI], 14–42 months), 22 (81%) patients progressed and/or died, and median PFS was 4 months (95% CI, 2–6 months). Factors associated with shorter median PFS on univariate analysis were high expression of BCL-W, defined as quartile 3–4 (3 vs 7 months, *p* = 0.001) (Fig. 1F–I) and GATA3/Th2 phenotype (3 vs 6 months, *p* = 0.04). No significant association between other baseline characteristics and PFS was observed. On multivariate analysis including both factors, the association with PFS was maintained only for high expression of BCL-W (hazard ratio [HR] 0.3, 95% CI 0.08–0.9, *p* = 0.04).

At most recent follow-up, 17 (63%) patients died, and median OS was 6 months (95% CI, 1–12 months). Factors associated with shorter OS on univariate analysis were high BCL-W expression (4 months vs not reached) (Fig. 1F–I), lack of consolidation with SCT (4 months vs not reached, *p* = 0.001), and GATA3/Th2 phenotype (4 vs 15 months, *p* = 0.03). No other significant association between other baseline characteristics and OS was observed.

On multivariate analysis, only lack of consolidation with SCT maintained a significant association with OS (HR 0.1, 95% CI 0.01–0.9, *p* = 0.04).

In this study, a higher expression of BCL-2A1 was observed in previously treated patients as compared to previously untreated patients. BCL-2A1 represents a common mechanism of acquired chemo-resistance, and its upregulation has been previously described in peripheral blood-derived CLL cell cultures treated with fludarabine [11, 12]. Further functional studies aimed at assessing whether its inhibition may decrease chemo-refractoriness in PTCL-NOS patients are warranted.

In our analysis, a higher BCL-2 expression was observed among patients with a GATA3/Th2 phenotype. Serum levels of Th1 and Th2-type effector cells-related cytokines (IL2, IFNγ, IL4, IL10, IL5, IL6, IL1β, TNFα, IL8) and soluble receptors (sIL2R, sIL6R) and BCL-2 proteins levels have been previously measured in a group of healthy subjects, showing an association between GATA3/Th2 phenotype and higher levels of BCL-2 [13]. These findings suggest that further investigation of the efficacy of BCL-2 inhibitors, such as venetoclax, in patients with PTCL-NOS and GATA3/Th2 phenotype may help identify better treatment strategies for this subgroup with otherwise very poor outcome.

Of interest, independent of GAT3/Th2 phenotype, increased BCL-W expression was associated with shorter PFS in our study. Functional studies aimed at confirming the negative prognostic impact played by BCL-W expression in PTCL-NOS observed in this study are warranted, while functional dependence on BCL-W for PTCL-NOS has yet to be proven [14].

In our analysis, the negative prognostic impact of BCL-W expression on OS was trumped by the use of SCT as a subsequent consolidation strategy. However, PTCL-NOS patients are frequently SCT-ineligible or experience sub-optimal outcomes with SCT, due to inhability to achieve CR before conditioning: novel agents, able to increase CR rate in these patients, are therefore desperately needed. Low doses of navitoclax have been investigated in combination with venetoclax in patients with relapsed or refractory T-cell acute lymphoblastic leukemia/lymphoma, showing better tolerability and an impressive 56% CR rate [15]. The development of more specific and safer BCL-W inhibitors and their investigation in PTCL pre-clinical models is needed.

We acknowledge the multiple limitations of this study, including its single center and retrospective nature, and its relatively small sample size, and lack of BH3 profiling data.

While clinical trials investigating the safety and efficacy of BCL-2 inhibition in PTCL-NOS are ongoing, these results suggest that concomitant BCL-W inhibition may be beneficial, and functional studies aimed at confirming these findings are highly needed.

## Supplementary information

Supplementary Figure 1

Supplementary Figure 2

Supplementary Legends

## Data Availability

The data that support the findings of this study are available from the corresponding author upon reasonable request.
