# Supplementary figures and images for: BCL-W expression associates with poor outcome in patients with peripheral T-cell lymphoma not otherwise specified
Source: Blood Cancer J. 2021 Sep 16;11(9):153. doi: 10.1038/s41408-021-00549-6 (PMC8445996; doi:10.1038/s41408-021-00549-6)

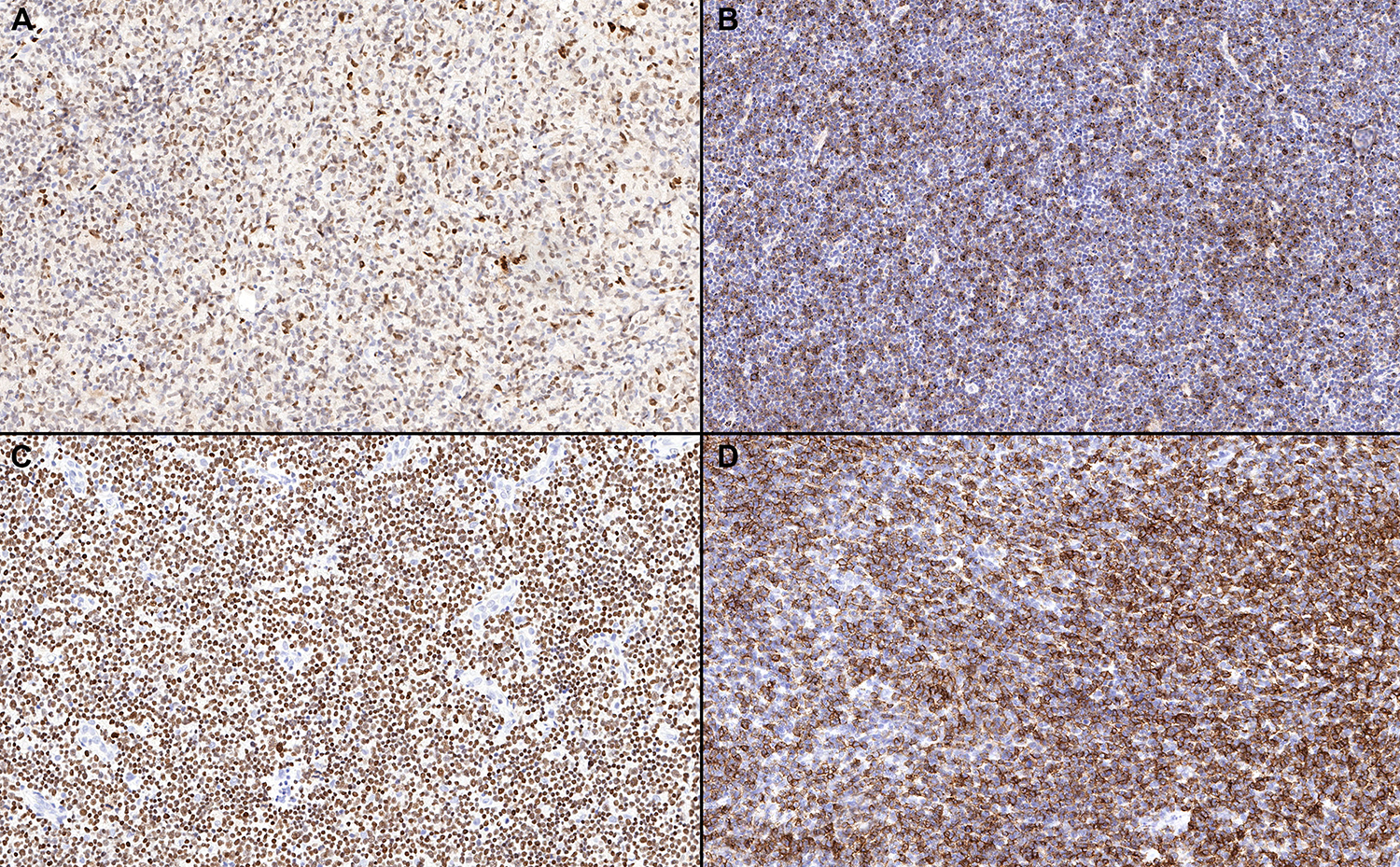

Supplement: Supplementary file 1 — Supplementary Figure 1 [file 41408_2021_549_MOESM1_ESM.tif]

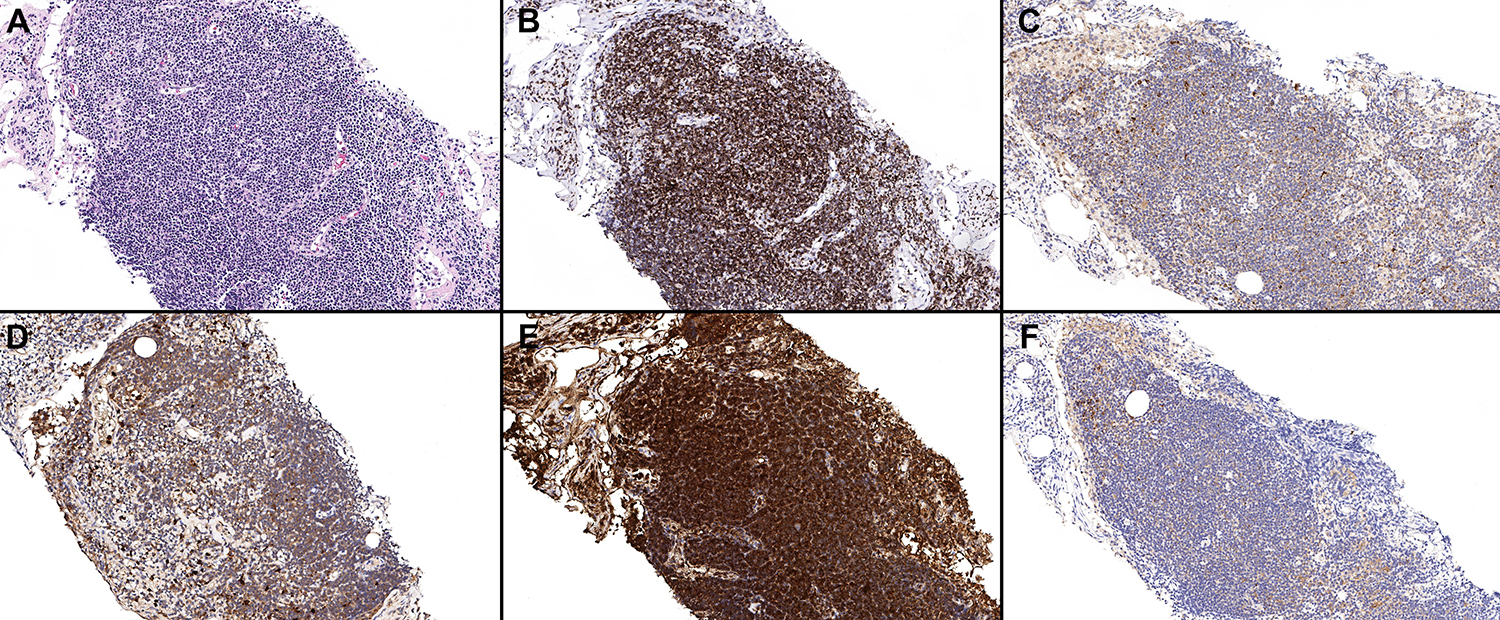

Supplement: Supplementary file 2 — Supplementary Figure 2 [file 41408_2021_549_MOESM2_ESM.tif]
