# Supplementary material for: BCL-W expression associates with poor outcome in patients with peripheral T-cell lymphoma not otherwise specified
Source: Blood Cancer J. 2021 Sep 16;11(9):153. doi: 10.1038/s41408-021-00549-6 (PMC8445996; doi:10.1038/s41408-021-00549-6)
Supplement: Supplementary file 3 — Supplementary Legends [file 41408_2021_549_MOESM3_ESM.docx]

**Supplementary Figure 1. Determination of cell-of-origin by immunohistochemistry according to Amador algorithm.**

A-B) Expression of TBX21 (A) and CXCR3 (one of the downstream targets of TBX21)(B) in ≥ 20% of the lymphoma cells in cases classified as TBX21/Th1 phenotype (C-D) Expression of GATA3 (C) and CCR4 (one of the downstream targets of GATA3) (D) in ≥ 50% of the lymphoma cells in cases classified as GATA3/Th2 phenotype.

**Supplementary Figure 2. Baseline expression of BCL-2 family proteins in PTCL NOS by Immunohistochemistry.**

A) Sections of a lymph node core biopsy with a diffuse effactement of the nodal architecture by small to intermediate atypical lymphocytes (H&E, 200x) ; B) BCL-2 immunostain shows strong expression in 100% of lymphoma cells (200x); C) MCL-1 immunostain shows heterogeneous and strong cytoplasmic positivity in 20% of lymphoma cells (200x); D) BCL-2A1 immunostain shows diffuse and strong cytoplasmic expression in 40% of lymphoma cells (200x); E) BCL-W immunostain shows diffuse and strong cytoplasmic positivity in 100% of lymphoma cells (200x); F) BCL-Xl immunostain shows heterogeneous and intense/moderate expression in 10% of the lymphoma cells (200x).
